# “It’s no use going back to yesterday”

**DOI:** 10.1308/rcsann.2024.0115

**Published:** 2025-01-01

**Authors:** B Rogers

**Figure:**
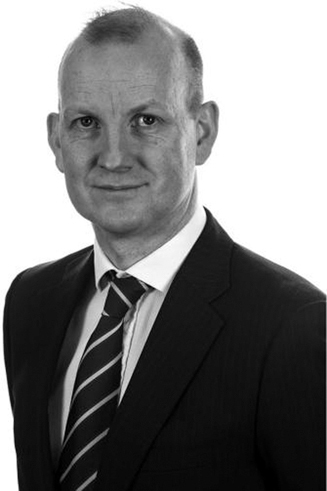


A lay person may consider surgery logical and simple to understand, a clinical problem with an operation to treat it. The reality, as all surgeons (should) know, is far from simple. Most surgical pathologies have numerous options for ‘when’, ‘how’, ‘who’ and ‘with what’. The evidence for each of these facets varies both in time and place. What is valid practice in one year can quickly become historical. Therefore, numerous profound ‘macro’ questions exist. Does the practice in one country translate fully to that in another? How does surgical practice adapt to changes in chronic disease epidemiology? Is the clinical evidence from privately or commercially funded institutions comparable to nationally funded ones? How translatable is research from a small to a large hospital? How do we prioritise the environmental impact of clinical surgery with new innovations, clinical risk and fiscal restrictions? How do we best train the surgeons of tomorrow and address a growing clinical caseload? How do we ensure surgeons fulfil a sustainable career without burnout?

These are only some of the important questions that surgeons, hospitals, professional bodies and governments strive to address. As we enter 2025, the *Annals* continues aiming to augment the evidence base for these problems that affect us all – either as surgeons or members of society. Furthermore, this highlights the wider question as to where surgeons consider their prime responsibility: to society, the profession or the individual. In short, to whom are a surgeon’s *prima facie* duties?

This edition of *Annals* includes reviews of quality of life following extensive pelvis surgery for colorectal cancer, dedicated trauma beds in recovery, plastic surgery exposure and awareness and a national review of emergency testicular fixation. A study I would like to highlight is by Chahal and Matwala, a systematic review that highlights the prevalence of burnout in orthopaedic surgeons to be approaching 50%.^1^ The relevance and importance of this study, to all surgeons, should not be underestimated.

Being a pan-specialty surgical journal, the *Annals* continues to be well placed to explore some of the wider questions by means of original research and academic debate. Published research provides a quantum of evidence to support or refute an aspect of surgery. Interpretations of evidence may vary and change, but innovation and change are true certainties. The *Annals* has had a very successful year. The journal transitioned to fully Open Access in January 2024, a move in line with publishing industry trends and the increasing demand for freely accessible research content. Consequently, usage has increased by more than 50% to almost 1m annual page views, with all content being immediately available in the public domain upon publication, driving reach, article downloads and citations.

As we commence 2025, change is the one obvious constant in surgical practice. Evaluating the evidence in a changing world, which the *Annals* aims to afford, becomes increasingly important. As a famous author from my alma mater once wrote “It’s no use going back to yesterday, because I was a different person then.”^2^
